# The relationship of PROMIS physical function scores and healthcare resource utilization in patients treated for chronic low back pain

**DOI:** 10.1016/j.inpm.2024.100522

**Published:** 2024-11-14

**Authors:** Andre Hejazi, Connor Willis, Xiangyang Ye, Jim Youssef, Chip Moebus, Ben Goss, Bryan Cornwall, Darrel Brodke, Zachary L. McCormick, Kenneth Schaecher, Diana Brixner

**Affiliations:** a Department of Pharmacotherapy, College of Pharmacy, University of Utah, Salt Lake City, UT, USA; b Mainstay Medical, San Diego, CA, USA; c Department of Orthopaedics, School of Medicine, University of Utah, Salt Lake City, UT, USA; d Department of Physical Medicine and Rehabilitation, School of Medicine, University of Utah, Salt Lake City, UT, USA; e University of Utah Health Plans, Salt Lake City, UT, USA

**Keywords:** Mechanical chronic low back pain, Chronic low back pain, Low back pain, Real-world evidence, Healthcare charges, Healthcare resource utilization, Non-surgical management

## Abstract

**Background context:**

Patients with mechanical chronic lower back pain (CLBP) have few durable treatment options for their condition and thus suffer decreased productivity and have higher healthcare resource utilization (HRU) compared to patients without CLBP. The economic burden of treatment and ongoing care for CLBP is considerable, with healthcare spending in 2016 estimated at $134.5 billion in the United States.

**Purpose:**

This study aims to assess the correlation between patient-reported physical function scores and HRU in patients treated for mechanical CLBP.

**Study design:**

This was a retrospective cohort study within a university-based health system.

**Patient sample:**

Patients with a diagnosis of mechanical CLBP from 2015 through 2020 (index date) who were non-surgical candidates at baseline were included in this study. To ensure the presence of *chronic* low back pain, patients were required to have encounters between 6 and 12 months as well as between 12 and 24 months following the date of CLBP diagnosis.

**Outcome measures:**

Collected data variables included patient baseline characteristics, Patient-Reported Outcomes Measurement Information System - Physical Function (PROMIS-PF) scores, pharmacologic and non-pharmacologic therapies, HRU, and healthcare charges between January 2015 through December 2022.

**Methods:**

PROMIS-PF scores were converted to numerical categories ranging from 0 to 3, with Category 0 representing the lowest physical function and Category 3 the highest physical function. Patients were more broadly stratified into Low Physical Function (Low-PF) (Category 0–1) or High-Physical Function(High-PF) (Category 2–3) cohorts. HRU was compared between the Low-PF and High-PF cohorts using linear regression analyses. A mixed-effects regression analysis comparing Low-PF and High-PF patients was performed to model the relationship between patient-reported physical function and healthcare charges. The model is able to estimate charges for a base-case patient and can be adjusted to include patient-specific characteristics.

**Results:**

A total of 2765 patients were included in this study, mean age was 50.1 (SD:17.7) years old, 23.6 % were 65 years or older, 68.4 % were female, and 85.3 % were white. Median healthcare charges by PROMIS-PF categories for Year-1 were highest for Category 0 patients ($14,028 [IQR: $5190-38,289]) and lowest for Category 3 ($5352 [IQR: $2417-14,470]). Patients in the Low-PF cohort showed significantly higher rates of all-cause, inpatient stays, outpatient visits, and emergency department (ED) visits compared to High-PF patients. The mixed effects regression model estimated cumulative healthcare charges to be > 2-fold higher for a base-case patient in the Low-PF cohort compared to High-PF. A small portion of patients (n = 14) failed treatment strategies and went on to receive CLBP-surgery despite not having surgical indications at baseline. Median healthcare charges from the 3-month period surrounding date of surgery were $59,809 (IQR: $46,057–85,484).

**Conclusions:**

Cumulative Year-1 healthcare charges were almost 3-fold higher in Low-PF patients compared to High-PF. The mixed effects regression model estimated cumulative 2-year charges to be over 2-fold higher for Low-PF compared to High-PF, in the base-case patient. There were significantly higher rates of all-cause inpatient, outpatient, and ED visits in the year following diagnosis of mechanical CLBP for Low-PF patients. Despite receiving treatment, some patients went on to receive costly surgical procedures over the course of follow-up.

## 1 Background

The lifetime prevalence of low back pain in the United States is 60–70 % [1], with 20 % of acute back pain cases developing into chronic low back pain (CLBP) [2]. Mechanical low back pain refers to back pain that arises intrinsically from the intervertebral discs, vertebral endplates, facet joints, sacroiliac joints, surrounding soft tissues, or a combination thereof [3]. Patients with mechanical CLBP, defined as persistent lower back pain symptoms for greater than 6 months without radicular symptoms, tend to have lower quality of life, decreased productivity, greater absenteeism from work, and higher healthcare resource utilization (HRU) than patients without CLBP [[4], [5], [6], [7]]. The diagnosis of mechanical CLBP is challenging and often requires a combination of clinical history, physical exam findings and diagnostic imaging studies to rule out surgical diagnoses or underlying pathophysiology that is the pain generator not amenable to surgical intervention. Historically this may have been referred to as “nonspecific” low back pain; however, clinicians are advancing in their ability to diagnose specific etiologies of CLBP [8].

Once patients are evaluated and found to be non-surgical candidates, treatment options in this patient population are variable. These include both pharmacological and non-pharmacological management, and often there is a complex treatment approach that combines both pharmacological and non-pharmacological treatments which are offered to such patients. Pharmacologic treatment often includes prescription of non-steroidal anti-inflammatory drugs and opioid medications, with 27 % of opioid-naïve patients newly diagnosed with LBP receiving opioid prescriptions [9]. Alternatively, non-pharmacologic management may include physical therapy, epidural injection, radiofrequency ablation for spinal joint or vertebral endplate denervation, peripheral nerve stimulation, spinal cord stimulation, and spinal surgery. The treatment regimen relies on the classification of CLBP with mechanical CLBP patients receiving a variety of treatment modalities that often lack durability or sustained beneficial outcomes. For those patients who fail such treatment options, and whose CLBP remains unresolved, high-cost surgical interventions are sometimes sought after by the patient and can lead to the same failure of durable pain relief seen with some non-operative spinal interventions [10].

Along with the humanistic toll experienced by patients with mechanical CLBP, the economic burden on our healthcare system of available treatments is considerable. Given this condition has historically had no available treatment that targets underlying cause, the continued cycle of palliation is a key driver of HRU [8]. In the United States, direct yearly spending on CLBP in 2016 was estimated at $134.5 billion [11]. However, with the added component of indirect costs including lost productivity, absenteeism, caregiving costs, etc., the total economic impact attributable to CLBP could be as high as $625 billion [11].

Given the magnitude of disability and cost associated with mechanical CLBP despite existing therapies, novel treatment modalities continue to be developed. As this occurs, it is important to evaluate not only the clinical outcomes, but the associated economic impact of emerging treatment options. To our knowledge, this is the first study to assess the relationship between patient-reported physical function scores and HRU in a real-world population of patients with mechanical CLBP and correlates the high cost of treating the highly disabled patients over time. We believe our findings could assist payers, policy makers, and other decision-makers to aid in estimating HRU and economic impact of new treatment options for mechanical CLBP based on changes in patient-reported physical function.

## 2 Methods

This was a retrospective cohort study assessing patient-reported physical function scores, HRU, and healthcare charges of patients diagnosed with mechanical CLBP within a university-based health system from January 1, 2015 through 12/31/2020.

### 2.1 Data source

Data was collected from an electronic health record (EHR) database within the University of Utah health system. The University of Utah health system is a major hub for healthcare in the geographic area and serves patients across socioeconomic levels. Extracted data included demographics, diagnostic codes (Table 8), procedure codes (Supplementary Table 1), outpatient visits, inpatient stays, and other healthcare encounters, and patient-reported outcome measurement information system-physical function (PROMIS-PF) scores. Healthcare charges were extracted using the university clinic and hospital billing database.

### 2.2 Study population

This study identified adult patients (18 years or older) with new onset mechanical CLBP via ICD-9 and ICD-10 diagnosis codes (see Table 8) between January 1, 2015 and December 31, 2020. Patients with a mechanical CLBP-related ICD code occurring within the prior year (2014) were not included in order to identify new CLBP cases only. To select for patients with *persistent* mechanical CLBP, a CLBP-related encounter was required both between 6 and 12 months as well as between 12 and 24 months post index diagnosis. Patients were required to have a baseline PROMIS-PF score documented in their charts within 6 months prior to date of index. Patients with cancer (other than skin cancer) and patients who were pregnant in the year prior to the index diagnosis were excluded. Additionally, patients with lumbar spine surgery, a diagnosis of FBSS, or post-laminectomy syndrome within 1-year pre- or 1-year post-index diagnosis were excluded (Table 8). Patients with inflammatory spondylopathies and other comorbid pain conditions at any point within the study period were also excluded (Table 8). Study variables for included patients were collected from January 1, 2015, through December 31, 2022, to ensure at least two years of follow-up for our cohort.

### 2.3 Study variables

Demographic and clinical characteristics included age, sex, race, ethnicity, BMI, and comorbidities were collected. Individual comorbidities were used as binary variables (present or absent) to better assess their specific impacts on outcomes. Specific variables were selected based on clinician expertise. HRU included all-cause and CLBP-related inpatient visits, outpatient visits, emergency department (ED) visits, and intensive care unit (ICU) stays. Only CLBP-related pharmacologic therapies and non-pharmacologic procedures were included. Non-pharmacologic procedures were identified using CPT codes (Supplementary Table 1). Pharmacologic therapies were obtained from structured medication order fields from the EHR. This study used healthcare charges, which are defined as the dollar amount set by healthcare administrators for a given service. The actual costs incurred by this patient population are not able to be published as they are proprietary to the university. This differs from healthcare costs, which reflect the actual amount the responsible party (usually third-party payer) must pay. Healthcare costs vary significantly based on the party incurring the expense and require access to a claims database. For this study, all-cause healthcare charges were extracted by an in-house data science team from the university's hospital billing data for included patients throughout follow-up. Technical/facility charges, inpatient medications, procedures, and physician/professional charges were included from the hospital billing data.

PROMIS-PF provides an efficient and precise measurement of a patient's physical functionality for a broad range of disabling conditions. This tool has been used extensively to determine the severity of CLBP, with lower physical function corresponding to higher disease severity [12]. Scores across all versions of the PROMIS-PF questionnaire were collected to estimate the severity of physical function for each patient across the study period. Scores across versions of the PROMIS-PF questionnaire (v1.0, v1.1, v1.2, and v2.0) were treated as comparable [13]. PROMIS-PF scores were converted into numerical categories as Category 0 (minimal physical function, <34.5), Category 1 (low physical function, 34.5 to <38.5), Category 2 (medium physical function, 38.5 to <47.2), or Category 3 (high physical function, ≥47.2). These thresholds were adapted from a previous analysis of pain categories by PROMIS-PF score in the arthritis or rheumatism population which significantly correlated the above thresholds to a real-world sample of patients [14].

For HRU and healthcare charge analyses, patients were stratified more broadly into either a Low Physical Function (Low-PF) cohort for patients in Category 0–1 or High Physical Function (High-PF) cohort for patients in Category 2–3.

### 2.4 Statistical analysis

#### 2.4.1 Baseline demographics

Baseline demographics for all included patients were stratified by PROMIS-PF categories (Category 0,1,2, and 3) and summarized using descriptive statistics. Mean/standard deviation (SD) and median/interquartile range (IQR) were reported for continuous variables. Frequency and percentage were reported for categorical variables. Statistical comparisons across physical function levels were performed using student-t and/or Wilcoxon rank sum test for continuous variables, and Chi-square test and/or Fisher's Exact test for categorical variables, where appropriate.

#### 2.4.2 Actual healthcare charges

Actual healthcare charges, stratified by PROMIS-PF categories, were summarized using descriptive statistics and reported as quarterly and yearly median/IQR charges from index date through the end of Year-2. All charges were converted to January 2020 USD using the Bureau of Labor Statistics Consumer Price Index [15,16].

#### 2.4.3 Healthcare resource utilization

Utilization of pharmacologic and non-pharmacologic therapies for the Low-PF and High-PF cohorts was reported as the count/percentage of patients receiving at least one given therapy in the 12 months following index date (Year-1). Statistical comparisons between the cohorts were performed using Chi-square tests.

Healthcare visits were assessed using mean per-patient-per-year (PPPY) visits from Year-1 for the Low-PF and High-PF cohorts. Low-PF patients were input into a generalized linear regression model (Poisson) and compared to High-PF patients as the reference cohort. Incidence rate ratios (IRR) for the Low-PF cohort were generated for each healthcare visit type as the model output and represent the average rate of utilization relative to the High-PF reference cohort. Length of stay (LOS) among patients with inpatient visits was calculated as mean days spent per visit in the 12 months following index. ANOVA regression was used to compare mean LOS per visit among Low-PF patients compared to High-PF patients as the reference cohort.

#### 2.4.4 Association between patient-reported physical function and healthcare charges

The association between PROMIS-PF scores and healthcare charges was assessed using a mixed-effects regression analysis. The outcome variable for the regression analysis was aggregated quarterly healthcare charges. Quarterly charges were log-transformed for the analysis, with predicted statistics reported at normal scale. In the first quarter, charges were classified by a patient's baseline PROMIS-PF score into Low or High-PF cohorts. The model accounted for repeated measurements, as each patient had up to eight quarterly observations of PROMIS-PF scores and charges. If no

PROMIS-PF score was captured in a given quarter, the score from the prior quarter was carried over. Individual patient identity was set as a random effect to account for the variation in scores and charges between patients over the two-year observation period. Fixed-effects included demographic characteristics and influential comorbidities, which were selected based on inclusion in the Charlson Comorbidity Index and expert clinical opinion. All comorbidities listed in Table 2 were controlled for in the model; only those associated with predicted charges and most relevant to the outcomes were reported in the regression result table (Table 7). Age was the only continuous variable; all other demographics and comorbidities were treated as categorical or dichotomous variables. All analyses were performed using STATA version 17, and statistical significance was set at a threshold of p < 0.05.

#### 2.4.5 Effect of patient characteristics on estimated healthcare charges

Incorporating the impact of a patients' demographic and comorbidities provides a more accurate estimate of healthcare charges. To estimate the effect of patient characteristics on quarterly charges, an algorithm was developed based on the mixed-effects regression analysis described above. The algorithm includes base-case (age = cohort median, BMI<25, Female, White, Non-Hispanic, and no comorbidities) quarterly charges for the High-PF, Low-PF, and overall cohort for Q1-Q8 in the first 8 rows. Q1 served as the reference, with the following quarters shown as the respective difference from Q1. The reference value for age was each respective cohort's median age. To apply this to a patient, the difference between the patient's age and median cohort age would be multiplied by the *charge* per *1-year increase* value and added to each quarterly charge. Similarly, the charge estimate associated with race, ethnicity, and comorbidities is added to each quarterly charge to estimate overall healthcare charges.

### 2.5 Charges associated with CLBP-related surgery

This study sought to capture patients with mechanical CLBP who were *not* candidates for surgery at diagnosis of CLBP. Non-surgical candidates were identified based on the included ICD codes (see Table 8) and by excluding individuals who received surgical interventions in the year prior to or post index date. Whether or not patients did eventually pursue surgical interventions over the course of follow-up was assessed as an exploratory measure and determined by the presence of relevant surgical CPT codes (Supplementary Table 1) beyond the 1-year post index time point. Charges in the 3-month window around date of surgery were described as mean/SD and median/IQR for this group of patients.

## 3 Results

A total of 2765 patients were included in this study. Patient inclusion flow diagram can be found in Supplementary Fig. 1. Based on baseline PROMIS-PF scores, 366 patients (13 %) were classified as Category 0, 376 (14 %) as Category 1, 1066 (39 %) as Category 2, and 957 (35 %) as Category 3 (Table 1).

**Table 1:** Cohort designations by baseline PROMIS-PF scores (n = 2765).

Physical functionality	PROMIS-PF score	PROMIS-PF category	n (%)	PF cohort	n (%)
Minimal	<34.5	0	366 (13 %)	Low-PF^a^	742 (27 %)
Low	34.5 - <38.5	1	376 (14 %)		
Medium	38.5 - <47.2	2	1066 (39 %)	High-PF^b^	2023 (73 %)
High	≥47.2	3	957 (35 %)		

High-PF, high-physical function; Low-PF, low-physical function; ODI, Oswestry Disability Index.

a Low-PF cohort (PROMIS-PF score <38.5) roughly translates to ODI scores >37.571.

b High-PF cohort (PROMIS-PF score ≥38.5) roughly translates to ODI scores ≤37.571.

**Table 2:** Baseline demographics by PROMIS-PF category.

	Overall	Category 0	Category 1	Category 2	Category 3
	(N = 2765)	(N = 366)	(N = 376)	(N = 1066)	(N = 957)
**Age**					
Mean (SD)	50.1 (17.7)	55.9 (18)	54.8 (17.6)	49.7 (18)	46.5 (16.4)
<65 years, n (%)	2113 (76.4)	254 (69.4)	258 (68.6)	817 (76.6)	784 (81.9)
≥65 years, n (%)	652 (23.6)	112 (30.6)	118 (31.4)	249 (23.4)	173 (18.1)
**BMI categories, n (%)**					
<25	903 (32.7)	113 (30.9)	93 (24.7)	344 (32.3)	353 (36.9)
25 - <30	779 (28.2)	95 (26)	99 (26.3)	290 (27.2)	295 (30.8)
>=30	1075 (38.9)	157 (42.9)	184 (48.9)	426 (40)	308 (32.2)
Missing	8 (0.3)	1 (0.3)	0 (0)	6 (0.6)	1 (0.1)
**Sex, n (%)**					
Female	1892 (68.4)	258 (70.5)	257 (68.4)	756 (70.9)	621 (64.9)
Male	873 (31.6)	108 (29.5)	119 (31.6)	310 (29.1)	336 (35.1)
**Race, n (%)**					
White	2359 (85.3)	317 (86.6)	323 (85.9)	926 (86.9)	793 (82.9)
Black	42 (1.5)	5 (1.4)	10 (2.7)	12 (1.1)	15 (1.6)
Asian	67 (2.4)	10 (2.7)	6 (1.6)	25 (2.3)	26 (2.7)
Other	274 (9.9)	31 (8.5)	36 (9.6)	90 (8.4)	117 (12.2)
Unknown	23 (0.8)	3 (0.8)	1 (0.3)	13 (1.2)	6 (0.6)
**Ethnicity, n (%)**					
Non-Hispanic	2444 (88.4)	329 (89.9)	336 (89.4)	952 (89.3)	827 (86.4)
Hispanic/Latino	261 (9.4)	28 (7.7)	32 (8.5)	89 (8.3)	112 (11.7)
Unknown	60 (2.2)	9 (2.5)	8 (2.1)	25 (2.3)	18 (1.9)
**Comorbidities, n (%)**					
Anxiety	865 (31.3)	141 (38.5)	130 (34.6)	350 (32.8)	244 (25.5)
Depression	1058 (38.3)	190 (51.9)	193 (51.3)	414 (38.8)	261 (27.3)
Bipolar	110 (4)	21 (5.7)	17 (4.5)	57 (5.3)	15 (1.6)
Schizophrenia	8 (0.3)	1 (0.3)	0 (0)	6 (0.6)	1 (0.1)
Hypertension	975 (35.3)	188 (51.4)	171 (45.5)	366 (34.3)	250 (26.1)
Obesity	568 (20.5)	93 (25.4)	109 (29)	217 (20.4)	149 (15.6)
Hypothyroidism	352 (12.7)	69 (18.9)	49 (13)	148 (13.9)	86 (9)
Coagulopathy	48 (1.7)	13 (3.6)	14 (3.7)	15 (1.4)	6 (0.6)
Myocardial infarction	126 (4.6)	42 (11.5)	25 (6.6)	39 (3.7)	20 (2.1)
Congestive heart failure	169 (6.1)	58 (15.8)	46 (12.2)	47 (4.4)	18 (1.9)
PVD	140 (5.1)	44 (12)	36 (9.6)	41 (3.8)	19 (2)
Cerebrovascular	138 (5)	34 (9.3)	26 (6.9)	46 (4.3)	32 (3.3)
Dementia	21 (0.8)	12 (3.3)	2 (0.5)	5 (0.5)	2 (0.2)
COPD	668 (24.2)	124 (33.9)	113 (30.1)	266 (25)	165 (17.2)
Rheumatic disease	173 (6.3)	43 (11.7)	32 (8.5)	75 (7)	23 (2.4)
Peptic ulcer disease	28 (1)	8 (2.2)	0 (0)	11 (1)	9 (0.9)
Mild liver disease	141 (5.1)	27 (7.4)	28 (7.4)	55 (5.2)	31 (3.2)
Diabetes, uncomplicated	213 (7.7)	38 (10.4)	48 (12.8)	72 (6.8)	55 (5.7)
Diabetes, complicated	226 (8.2)	59 (16.1)	38 (10.1)	89 (8.3)	40 (4.2)
Hemiplegia	45 (1.6)	19 (5.2)	13 (3.5)	11 (1)	2 (0.2)
Renal disease	207 (7.5)	54 (14.8)	42 (11.2)	72 (6.8)	39 (4.1)
Severe liver disease	27 (1)	7 (1.9)	9 (2.4)	8 (0.8)	3 (0.3)
Metastatic cancer	1 (0)	0 (0)	0 (0)	1 (0.1)	0 (0)
AIDS/HIV	45 (1.6)	6 (1.6)	7 (1.9)	21 (2)	11 (1.1)
**CCI, mean (SD)**	1.1 (1.9)	2.0 (2.4)	1.6 (2.2)	1.1 (1.8)	0.6 (1.3)

BMI, body mass index; COPD, chronic obstructive pulmonary disease; CCI, Charlson Comorbidity Index; AIDS, acquired immunodeficiency syndrome; PVD, peripheral vascular disease; HIV, human immunodeficiency virus; SD, standard deviation.

### 3.1 Baseline demographics

In the overall cohort (n = 2765), mean age was 50.1 (SD:17.7) years old, 23.6 % were 65 years or older, 68.4 % were female, and 85.3 % were white (Table 2). Depression, hypertension, and anxiety were the most prevalent comorbidities at 38.3 %, 35.3 %, and 31.3 %, respectively.

Stratified by baseline PROMIS-PF categories, mean age was highest for the Category 0 cohort (55.9 years, SD: 18.0) and lowest for Category 3 (46.5 years, SD: 16.4). More patients were obese (BMI>30) in the Category 0 cohort compared to Category 3 (42.9 % vs 32.2 %). Category 0 patients tended to have higher comorbidity rates than the Category 3, with notable increases in anxiety (38.5 % vs 25.5 %), depression (51.9 % vs 27.3 %), and hypertension (51.4 % vs 26.1 %). All comparisons between PROMIS-PF categories were statistically significant (p < 0.001).

### 3.2 Actual healthcare charges

**Fig. 1:**
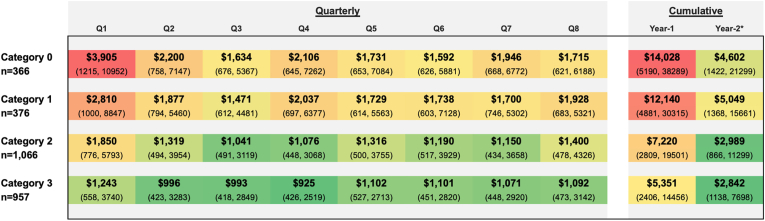
Quarterly and cumulative median healthcare charges by PROMIS-PF categories Actual quarterly and cumulative healthcare charges over the course of 2-year follow-up are shown as group medians with IQR in parentheses, stratified by baseline PROMIS-PF categories. Charges are color coded from lowest (green) to highest (red) for quarterly and cumulative sections. IQR, interquartile range.

Median healthcare charges by PROMIS-PF category in the first quarter (Q1) following index date were highest among patients in the Category 0 cohort ($3905 [IQR: $1215-10,952]), followed by Category 1 ($2810 [IQR: $1000–8847]), Category 2 ($1850 [IQR: $776-5793]), and Category 3 ($1243 [IQR: $558-3740]) (Fig. 1). A notable spike in charges was observed in Q1 for both Category 0 and 1 patients.

**Fig. 2:**
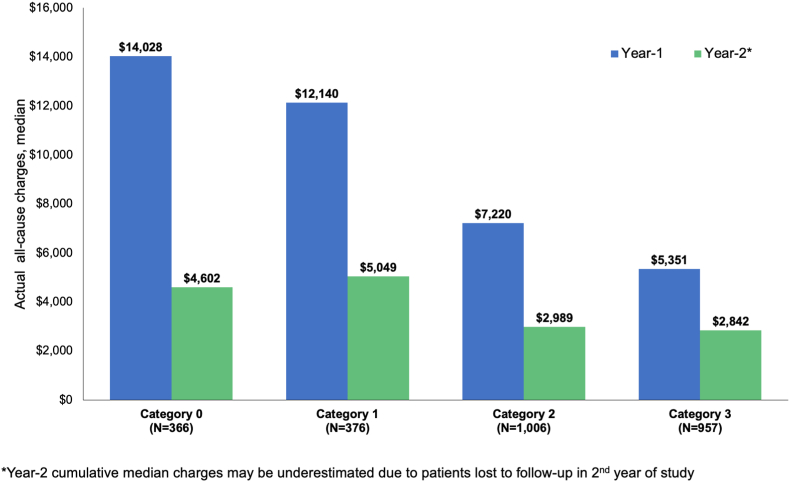
Cumulative median healthcare charges by PROMIS-PF categories Actual cumulative healthcare charges for Year-1 and Year-2 following index date are shown as medians with interquartile ranges, stratified by baseline PROMIS-PF categories. IQR, interquartile range.

Median cumulative charges in the first- and second-year following index (Year-1 and Year-2) are shown in Fig. 2. Year-1 charges increased with declining physical function, with roughly 3-fold higher charges among Category 0 patients compared to Category 3 ($14,028 vs. $5351). Charges followed a similar trend in Year-2 but were lower across all cohorts compared to Year-1.

### 3.3 Healthcare resource utilization

#### 3.3.1 Non-pharmacologic procedures

Non-pharmacologic procedure utilization in Year-1 for patients in the Low-PF (Category 0–1, n = 742) and High-PF (Category 2–3, n = 2023) cohorts are shown in Table 3. Physical therapy had the highest utilization overall, with 30.9 % of Low-PF patients and 35.9 % of High-PF patients receiving at least one session. Acupuncture and massage showed similar trends of higher utilization with higher physical function. Procedures showing decreased utilization from the Low-PF to High-PF cohort were EMG (0.8 % vs 0.2 %, p < 0.01), ketamine infusions (5.3 % vs 2.2 %, p < 0.01), sympathetic nerve blocks (3.8 % vs 1.8 %), p < 0.01) and psychotherapy (10.9 % vs 6.5 %, p < 0.01). There was no difference in utilization between the Low-PF and High-PF groups for RFA (1.1 % vs 0.9 %, p = 0.74) and epidural injections (1.3 % vs 1.2 %, p = 0.82).

**Table 3:** Nonpharmacologic procedures by Physical Function cohort, Year-1.

Procedures, n (%)	Low-PF	High-PF	p-value
	N = 742	N = 2023	
Physical therapy	229 (30.9 %)	726 (35.9 %)	**0.01**^b^
Psychotherapy	81 (10.9 %)	132 (6.5 %)	**<0.01**^b^
Biofeedback	59 (8.0 %)	125 (6.2 %)	0.10^b^
X-ray	50 (6.7 %)	126 (6.2 %)	0.63^b^
Ketamine infusion	39 (5.3 %)	44 (2.2 %)	**<0.01**^b^
Trigger point injections	32 (4.3 %)	58 (2.9 %)	0.06^b^
Sympathetic nerve block	28 (3.8 %)	37 (1.8 %)	**<0.01**^b^
CT scan	24 (3.2 %)	34 (1.7 %)	**0.01**^b^
Botox	20 (2.7 %)	33 (1.6 %)	0.07^b^
MRI	20 (2.7 %)	29 (1.4 %)	**0.03**^b^
Epidural injection	10 (1.3 %)	25 (1.2 %)	0.82^b^
RFA	8 (1.1 %)	19 (0.9 %)	0.74^b^
EMG	6 (0.8 %)	3 (0.1 %)	**<0.01**^b^
Acupuncture	2 (0.3 %)	18 (0.9 %)	0.13^a^
Basivertebral nerve ablation	2 (0.3 %)	4 (0.2 %)	0.66^a^
Massage	1 (0.1 %)	20 (1.0 %)	**0.02**^a^
Occupational therapy	0 (0.0 %)	2 (0.1 %)	1.00^a^
Intrathecal pain pump	0 (0.0 %)	0 (0.0 %)	NA

CT, computed tomography; EMG, electromyography; MRI, magnetic resonance imaging; RFA, radiofrequency ablation; High-PF, high-physical function; Low-PF, low-physical function.

Counts/percentages represent number of patients receiving at least one procedure in the first year following CLBP diagnosis date.

a Fisher's exact.

b Chi-square.

#### 3.3.2 Pharmacologic therapies

Medication orders in Year-1 for the Low-PF and High-PF cohorts are shown in Table 4. Medication classes showing notably higher utilization in the Low vs High-PF groups were opioids (56 % vs 39 %, p < 0.01), acetaminophen (36 % vs 22 %, p < 0.01), anticonvulsants (43 % vs 24 %, p < 0.01), antidepressants (48 % vs 39 %, p < 0.01), and NSAIDs (48 % vs 40 %, p < 0.01). There was no difference in utilization between Low-PF and High-PF groups for musculoskeletal relaxants (19 % vs 20 %, p = 0.77).

**Table 4:** Medication orders by Physical Function cohort, Year-1.

Medications, n (%)	Low-PF	High-PF	p-value^a^
	N = 742	N = 2023	
Opioids	413 (56 %)	786 (39 %)	**<0.01**
NSAIDS	356 (48 %)	816 (40 %)	**<0.01**
Antidepressants	355 (48 %)	780 (39 %)	**<0.01**
Anticonvulsants	316 (43 %)	492 (24 %)	**<0.01**
Acetaminophen^b^	264 (36 %)	437 (22 %)	**<0.01**
Musculoskeletal relaxants	143 (19 %)	400 (20 %)	0.77

High-PF, high-physical function; Low-PF, low-physical function; NSAIDS, non-steroidal anti-inflammatory drugs.

Counts/percentages represent number of patients receiving at least one medication in the first year following CLBP diagnosis date.

a Chi-square.

b Includes combination drugs with acetaminophen (non-opioid).

#### 3.3.3 Healthcare visits

Patients in the Low-PF cohort showed on average a 2.2 times higher rate (95 % CI: 1.8–2.6, p < 0.01) of all-cause inpatient hospitalizations compared to the High-PF cohort. On average, the length of stay (LOS) for inpatient visits were 2.9 days longer (CI: 1.3–4.4, p < 0.01) per visit for Low-PF patients compared to High-PF patients (Table 6). The rate of outpatient visits (IRR = 1.2, CI: 1.1–1.3, p < 0.01) and ED visits (IRR = 1.9, CI: 1.5–2.4, p < 0.01) were also higher in the Low-PF cohort.

**Table 5:** Regression analysis of healthcare visits in Low-PF patients, Year-1.

Visit type	Low-PF (n = 742)		
	IRR	95 % CI	p-value^a^
**All-cause**			
Inpatient stays	2.2	(1.8–2.6)	**<0.01**
Outpatient visits	1.2	(1.1–1.3)	**<0.01**
ED visits	1.9	(1.5–2.4)	**<0.01**
ICU stays^b^	NA	NA	NA
Urgent care visits	0.9	(0.8–1.0)	0.11
**CLBP-related**			
Inpatient stays	2.8	(0.6–14.5)	0.21
Outpatient visits	0.9	(0.7–1.0)	0.11
ED visits	1.8	(1.0–3.3)	**0.04**
ICU stays^b^	NA	NA	NA
Urgent care visits	0.6	(0.3–1.1)	0.08

CI, confidence interval; ED, emergency department; ICU, intensive care unit; IRR, incidence rate ratio; High-PF, high-physical function; Low-PF, low-physical function.

IRR represents average rate of visits in Year-1 for Low-PF patients relative to High-PF reference cohort (n = 2023); High-PF IRR = 1.

a Poisson regression.

b Too few visits to determine.

**Table 6:** Regression analysis of inpatient LOS in Low-PF patients, Year-1.

Inpatient LOS, days	Low-PF (n = 256) a		
	Coefficient	95 % CI	p-value^b^
All-cause	2.9	(1.3–4.4)	**<0.01**
CLBP-related	1.7	(-0.8 - 4.1)	0.13

CI, confidence interval; LOS, length of stay; High-PF, high-physical function; Low-PF, low-physical function.

Coefficient represents average days spent per inpatient visit in Year-1 for Low-PF patients relative to High-PF reference cohort (n = 439); High-PF Coefficient = 0.

a Among subgroup of patients with at least one inpatient visit.

b ANOVA regression.

CLBP-related ED visits showed a similar trend to all-cause utilization, with 1.8 times higher utilization in the Low-PF cohort (CI: 1.0 - 3.3 p = 0.04) (Table 5). CLBP-related inpatient visits were roughly 2.7 times higher (CI: 0.6–14.5), and outpatient visits were 0.9 times lower (CI: 0.7–1.0) in the Low-PF cohort compared to High-PF, though neither were statistically significant.

**Table 7:** Mixed-effect regression model of estimated healthcare charges from diagnosis through Year-2.

Variable	Overall (N = 2765)				Low-PF (N = 742)				High-PF (N = 2023)			
	Median age: 49.5				Median age: 56				Median age: 47.2			
	R^2^ = 0.325				R^2^ = 0.291				R^2^ = 0.367			
	Charge	95 % CI		P-value	Charge	95 % CI		P-value	Charge	95 % CI		P-value
	Estimate^a^	Lower	Upper		Estimate^a^	Lower	Upper		Estimate^a^	Lower	Upper	
**Base-case scenario**												
*Quarter: 1 (reference)*	1532	1335	1758	**<.001**	3020	2171	4203	**<.001**	1344	1153	1567	**<.001**
Quarter: 2	−545	−613	−473	**<.001**	−1220	−1449	−957	**<.001**	−454	−525	−378	**<.001**
Quarter: 3	−641	−701	−577	**<.001**	−1533	−1719	−1319	**<.001**	−512	−577	−441	**<.001**
Quarter: 4	−609	−671	−543	**<.001**	−1211	−1439	−950	**<.001**	−533	−596	−465	**<.001**
Quarter: 5	−567	−632	−496	**<.001**	−1421	−1623	−1189	**<.001**	−439	−511	−362	**<.001**
Quarter: 6	−583	−648	−513	**<.001**	−1452	−1654	−1220	**<.001**	−456	−526	−380	**<.001**
Quarter: 7	−568	−635	−497	**<.001**	−1342	−1561	−1089	**<.001**	−460	−531	−384	**<.001**
Quarter: 8	−542	−611	−467	**<.001**	−1471	−1675	−1235	**<.001**	−399	−476	−316	**<.001**
**Physical function**												
*High-PF (reference)*												
Low-PF	526	366	699	**<.001**	NA				NA			
**Age**												
*Cohort median (reference)*												
Charge per 1-year increase from median	1	−2	5	0.451	−14	−28	1	0.057	4	1	8	**0.024**
**Sex**												
*Female (reference)*												
Male	−138	−239	−29	**0.012**	−302	−704	169	0.196	−119	−220	−10	**0.034**
**Race:**												
*White (reference)*												
Black	176	−242	729	0.448	954	−643	3624	0.295	−41	−417	488	0.858
Asian	−149	−421	190	0.359	−1161	−1894	51	0.058	2	−288	370	0.993
Other	20	−186	258	0.858	216	−635	1370	0.658	−3	−202	231	0.977
Unknown	−166	−604	477	0.56	−944	−2231	2446	0.448	−113	−537	533	0.682
**Ethnicity**												
*Non-Hispanic (reference)*												
Hispanic/Latino	−174	−358	39	0.105	−75	−886	1044	0.878	−182	−356	23	0.079
Unknown	90	−258	534	0.642	−289	−1329	1392	0.681	141	−226	627	0.491
**Comorbidities**												
*None (reference)*												
Anxiety	205	67	354	**0.003**	562	7	1220	**0.048**	136	2	284	**0.047**
Depression	260	120	412	**<.001**	473	−63	1106	0.087	235	94	390	**0.001**
Bipolar	279	−9	622	0.058	515	−471	1883	0.346	207	−82	563	0.174
Schizophrenia	1005	−150	3123	0.104	−279	−2628	16145	0.922	1167	−1	3353	**0.05**
Hypertension	169	25	326	**0.02**	555	−21	1242	0.061	99	−42	255	0.177
Obesity	152	−4	325	0.056	189	−372	869	0.536	150	−11	330	0.07
Hypothyroidism	211	40	400	**0.014**	695	14	1528	**0.046**	111	−54	298	0.198
Coagulopathy	371	−72	950	0.109	871	−436	2838	0.226	351	−180	1124	0.227

R^2^ represents goodness-of-fit for model.

CI, confidence interval; High-PF, high-physical function; Low-PF, low-physical function.

Model is adjusted for demographic and comorbidities and shows quarterly charge predictions for Q1-Q8 in the first 8 rows for base-case patient (age = cohort median, BMI<25, Female, White, Non-Hispanic, and no comorbidities). All comorbidities listed in Table 2 were controlled for in the model; only those associated with predicted charges and most relevant to the outcomes were reported in the regression result table. Q1 served as the reference, with the following quarters shown as the respective difference from Q1. To factor in patient characteristics into prediction, add charge estimate for given variable to each quarterly charge. See Fig. 4 for examples of how to apply this model.

a Difference in charges ($USD) from respective *reference* value.

**Table 8:** ICD codes used for inclusion/exclusion criteria.

**Included ICD codes**	
**ICD-9**	
721.3	Lumbosacral spondylosis without myelopathy
721.9	Spondylosis NOS without myelopathy
722.1	Displacement of lumbar disc without myelopathy
722.52	Lumbar/lumbosacral disc degeneration
722.6	Disc degeneration NOS
722.93	Other and unspecified lumbar disc disorder
724.02	Spinal stenosis, lumbar region without neurogenic claudication
724.2	Lumbago
724.5	Backache
756.11	Lumbosacral spondylosis
**ICD-10**	
M43.00	Spondylolysis, site unspecified
M43.05	Spondylolysis, thoracolumbar region
M43.06	Spondylolysis, lumbar region
M43.07	Spondylolysis, lumbosacral region
M47.81	Spondylosis without myelopathy or radiculopathy
M47.816	Spondylosis without myelopathy or radiculopathy, lumbar region
M47.817	Spondylosis without myelopathy or radiculopathy, lumbosacral region
M47.819	Spondylosis without myelopathy or radiculopathy, site unspecified
M47.896	Other spondylosis, lumbar region
M47.897	Other Spondylosis, lumbosacral region
M47.899	Other spondylosis, site unspecified
M47.9	Spondylosis, unspecified
M48.061	Spinal stenosis, lumbar region without neurogenic claudication
M51.26	Other intervertebral disc displacement, lumbar region
M51.27	Other intervertebral disc displacement, lumbosacral region
M51.36	Other intervertebral disc degeneration, lumbar region
M51.37	Other intervertebral disc degeneration, lumbosacral region
M51.86	Other intervertebral disc disorders, lumbar region
M51.87	Other intervertebral disc disorders, lumbosacral region
M54.5	Low back pain
M54.50	Unspecified
M54.51	Vertebrogenic low back pain
M54.59	Other low back pain
M54.89	Other dorsalgia
M54.9	Dorsalgia, unspecified

**Excluded ICD codes**	
**Neuropathy/Neuropathic pain conditions (1-year pre and post-index)**	
**ICD-9**	
336.3	Myelopathy in other diseases classified elsewhere
336.9	Unspecified disease of spinal cord
721.4	Thoracic or lumbar spondylosis with myelopathy
721.41	Thoracic spondylosis with myelopathy, **lumbar** region
721.42	Spondylosis with myelopathy, lumbar/lumbosacral region
721.91	Spondylosis NOS; with myelopathy
722.7	Intervertebral disc disorder with myelopathy
722.7	Intervertebral disc disorder with myelopathy; unspecified region
722.73	Intervertebral disc disorder with myelopathy; lumbar region
**ICD-10**	
G99.2	Myelopathy in diseases classified elsewhere
G95.9	Disease of spinal cord, unspecified
M47.10	Other spondylosis with myelopathy, site unspecified
M47.16	Other spondylosis with myelopathy, lumbar region
M51.06	Intervertebral disc disorders with myelopathy, lumbar region
M51.07	Intervertebral disc disorders with myelopathy, lumbosacral region
**Radiculopathy (1-year pre- and post-index)**	
**ICD-9**	
724.3	Sciatica NOS
724.4	Thoracic or lumbosacral neuritis or radiculitis, unspecified
729.2	Neuralgia, neuritis, radiculitis, unspecified
53.1	Herpes zoster with unspecified nervous system complication
53.19	Herpes zoster with other nervous system complications
**ICD-10**	
B02.29	Other postherpetic nervous system involvement (radiculopathy)
M47.20	Other spondylosis with radiculopathy, site unspecified
M47.26	Other spondylosis with radiculopathy, lumbar region
M47.27	Other spondylosis with radiculopathy, lumbosacral region
M47.28	Other spondylosis with radiculopathy, sacral and sacrococcygeal region
M51.16	Intervertebral disc disorders with radiculopathy lumbar region
M51.17	Intervertebral disc disorders with radiculopathy, lumbosacral region
M54.10	Radiculopathy, site unspecified
M54.16	Radiculopathy, lumbar region
M54.17	Radiculopathy, lumbosacral region
M54.18	Radiculopathy, sacral and sacrococcygeal region
M54.4	Lumbago with sciatica
[M54.40] [M54.41] [M54.42]	
M54.3	Sciatica
[M54.30] [M54.21] [M54.32]	
**Spinal stenosis with claudication (1-year pre- and post-index)**	
**ICD-9**	
724.03	Spinal stenosis, lumbar region with neurogenic claudication
**ICD-10**	
M48.062	Spinal stenosis, lumbar region with neurogenic claudication
**Inflammatory spondylopathies (entire study time window)**	
**ICD-9**	
720	Ankylosing spondylitis and other inflammatory spondylopathies
720	Ankylosing spondylitis
720.1	Spinal enthesopathy
720.2	Sacroiliitis NEC
720.8	Other inflammatory spondylopathies
720.81	Inflammatory spondylopathies in diseases classified elsewhere
720.89	Inflammatory spondylopathies; other
720.9	Inflammatory spondylopathy NOS
722.93	Discitis, lumbar/lumbosacral
730.28	Unspecified osteomyelitis, other specified sites
730.98	Unspecified infection of bone, other specified sites
**ICD-10**	
M45.7	Ankylosing spondylitis of lumbosacral region
M45.8	Ankylosing Spondylitis sacral and sacrococcygeal region
M45.9	Ankylosing spondylitis of unspecified sites in spine
M46.00	Spinal enthesopathy, site unspecified
M46.06	Spinal enthesopathy, lumbar region
M46.07	Spinal enthesopathy, lumbosacral region
M46.08	Spinal enthesopathy, sacral and sacrococcygeal region
M46.09	Spinal enthesopathy, multiple sites in spine
M46.1	Sacroiliitis, NEC
M46.20	Osteomyelitis of vertebra, site unspecified
M46.26	Osteomyelitis of vertebra, lumbar region
M46.27	Osteomyelitis of vertebra, lumbosacral region
M46.28	Osteomyelitis of vertebra, sacral and sacrococcygeal region
M46.30	Infection of intervertebral disc (pyogenic), site unspecified
M46.36	Infection of intervertebral disc (pyogenic), lumbar region
M46.37	Infection of intervertebral disc (pyogenic), lumbosacral region
M46.38	Infection of intervertebral disc (pyogenic), site sacral and sacrococcygeal region
M46.39	Infection of intervertebral disc (pyogenic), multiple sites in spine
M46.8	Other inflammatory spondylopathies
M46.80	Other inflammatory spondylopathies, site unspecified
M46.86	Other specified inflammatory spondylopathies, lumbar region
M46.87	Other specified inflammatory spondylopathies, lumbosacral region
M46.88	Other specified inflammatory spondylopathies, sacral and sacrococcygeal region
M46.89	Other specified inflammatory spondylopathies, multiple sites in spine
M46.90	Unspecified inflammatory spondylopathy, site unspecified
M46.96	Unspecified inflammatory spondylopathy, lumbar region
M46.97	Unspecified inflammatory spondylopathy, lumbosacral region
M46.98	Unspecified inflammatory spondylopathy, sacral and sacrococcygeal region
M46.99	Unspecified inflammatory spondylopathy, multiple sites in spine
M46.46	Discitis, unspecified, lumbar region
M46.47	Discitis, unspecified, lumbosacral region
M46.48	Discitis, unspecified, sacral and sacrococcygeal region
M46.49	Discitis, unspecified, multiple sites in spine
M46.50	Other infective spondylopathies, site unspecified
M46.56	Other infective spondylopathies, lumbar region
M46.57	Other infective spondylopathies, lumbosacral region
M46.58	Other infective spondylopathies, sacral and sacrococcygeal region
M46.59	Other infective spondylopathies, multiple sites in spine
**Other comorbid pain conditions (entire study time window)**	
**ICD-9**	
338.3	Neoplasm related pain (acute) (chronic)
**ICD-10**	
G89.3	Neoplasm related chronic pain

### 3.4 Association between patient-reported physical function and healthcare charges

#### 3.4.1 Estimated charges for a reference patient using mixed-effect regression model

**Fig. 3:**
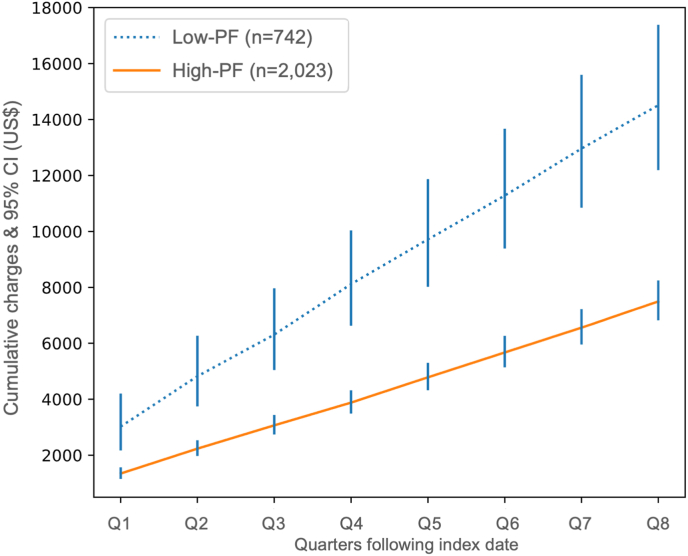
Estimated quarterly charges by Physical Function cohort Estimated cumulative charges (through Year-2) by quarter using estimates from regression model for a Low-PF and High-PF base-case patient. Base-case patient characteristics are the following: age = cohort median, BMI<25, Female, White, Non-Hispanic, and no comorbidities. CI, confidence interval; High-PF, high-physical function; Low-PF, low-physical function.

Estimated cumulative 2-year charges for the Low-PF and High-PF cohorts from Q1 to Q8, adjusted for demographic characteristics and comorbidities, are shown in Fig. 3. Importantly, these estimated charges represent a base-case scenario for a reference patient (age = cohort median, BMI<25, Female, White, Non-Hispanic, and no comorbidities). Estimated Q1 charges were >2-fold higher for Low-PF patients compared to High-PF ($3020 vs $1344) (Fig. 3). This trend continued through Q8, with estimated cumulative charges at Year-2 being roughly 2-fold higher for Low-PF patients compared to High-PF ($14,510 vs $7499) (Fig. 3).

#### 3.4.2 Estimated effect of patient characteristics on estimated healthcare charges

The *charge* per *1-year increase* in age was -$14 [CI: -$28, 1] (p = 0.057) for Low-PF patients and +$4 [CI: $1, 8] (p = 0.024) per quarter for High-PF. (Table 7). Male patients in both Low and High-PF groups were associated with decreased charges per quarter compared to females (Low: -$302 [CI: -$704, 169], p = 0.196; High: -$119 [CI: -$220, −10], p = 0.034). Higher quarterly charges were observed in patients who self-reported their race as Black, especially in the Low-PF cohort (+$954 [CI: -$643, 3624], p = 0.295); though these findings were not statistically significant. Anxiety and depression were associated with increases in quarterly charges within the Low-PF cohort (+$562 [CI: $7, 1220], p = 0.048 & +$473 [CI: -$63, 1106], p = 0.087; respectively) and the High-PF cohort (+$136 [CI: $2, 284], p = 0.047 & +$235 [CI: $94–390], p < 0.001; respectively).

The impact of the single variable of physical function on healthcare charges while controlling for demographic characteristics and selected comorbidities was assessed among the overall cohort (n = 2765). Low physical function was associated with an estimated +$526 [CI: $366, 699] (p < 0.001) increase in healthcare charges per patient per quarter or +$2104 increase per patient per year, when compared to High physical function (Table 7).

**Fig. 4:**
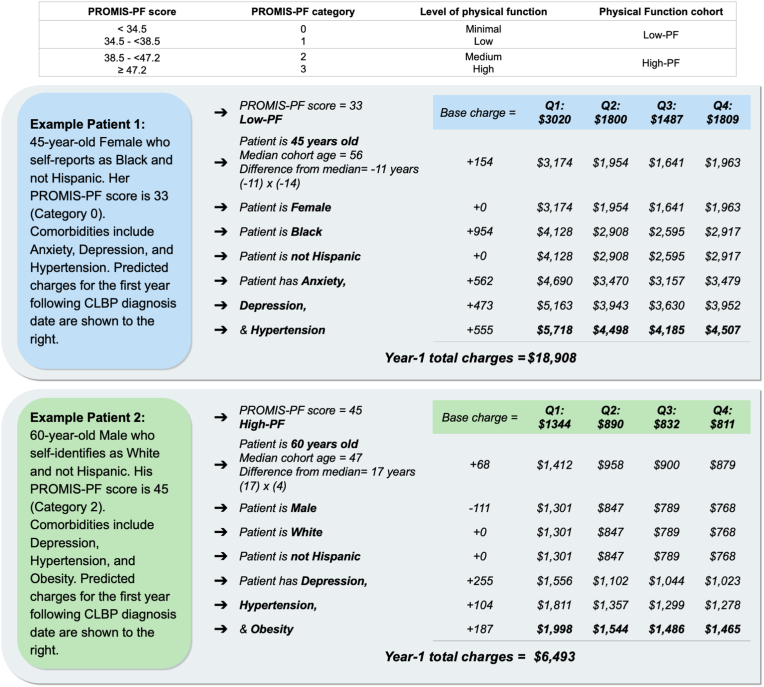
Application of Model Output Examples of how regression model output could estimate healthcare charges. Characteristics (age, sex, race, ethnicity, and comorbidities) and PROMIS-PF scores for 2 hypothetical ‘example’ patients are shown in boxes on the left. On the right, charges for Q1-Q4 following diagnosis are calculated using model charge estimates for each example patient. Quarterly base charges and corresponding charges related to patient characteristics taken from regression model out shown in Table 7. Case examples illustrate significant estimated charge differences between a patient with a Category 0 and Category 2 PROMIS-PF score. Of note, this model reflects the regression output from an observational single-site study and should not be used as a predictive tool. High-PF, high-physical function; Low-PF, low-physical function.

Examples of how the regression model output can be used to estimate healthcare charges for newly diagnosed patients with CLBP are shown in Fig. 4.

### 3.5 Charges associated with CLBP-related surgery

Despite not having surgical indications at baseline, a small group of patients (n = 14) within our cohort did eventually pursue CLBP-related surgery following the 1-year post index time point. Among this cohort of 14 patients, mean and median healthcare charges in the 3-month window around date of surgery were $69,114 (SD:$34,952) and $59,809 (IQR: $46,057–85,484), respectively.

## 4 Discussion

This study reports actual and estimated healthcare charges among non-surgical candidate patients with mechanical CLBP within an academic health system, stratified by patient-reported PROMIS-PF scores. Actual cumulative Year-1 charges were almost 3-fold higher for patients reporting minimal physical function (Category 0) compared to high physical function (Category 3) ($14,028 vs $5352). Further, this study demonstrated that charges continued to rise at a steady rate following initial Q1 spike throughout the 2-year follow-up period. Adjusted for patient demographics and comorbidities in our study, Low-PF was associated with a >2-fold increase in cumulative charges compared to High-PF over a two-year period. Additionally, we compared HRU between Low-PF and High-PF cohorts and demonstrated significantly higher rates of all-cause inpatient, outpatient, and ED visits in the first year following mechanical CLBP diagnosis among Low-PF patients. To our knowledge, this study is the first to both quantify the relationship between mechanical CLBP severity based on patient reported outcomes and healthcare charges/utilization and estimate charges based on a reliable patient-reported physical function scoring tool.

The healthcare charges used in this analysis reflect the amount billed by a health care professional and facilities for products and services provided to patients. Healthcare costs reflect the actual amount reimbursed to the health provider for service and varies significantly based on negotiated rates. The Centers for Medicare & Medicaid Services (CMS) publishes a cost-to-charge ratio (CCR) specific to each major health system which can be used to convert charges to costs to better reflect the actual healthcare spending [17]. The 2024 CCR for this health system is 0.426 (Operating CCR + Capital CCR), which is higher than the national average of 0.313 [17]. Applying our CCR to the actual Year-1 healthcare charges for the PROMIS-PF Category 0 cohort, the adjusted cost would be approximately $5976 ($14028 x 0.426). This adjusted cost is included as an example and should not be compared to healthcare costs outside of this specific health system. Overall, the charge data presented in this study should be interpreted based on the direction of change between the physical function cohorts instead of the absolute values.

The considerable economic burden of available therapy options for CLBP has been described previously [18,19]. A recent MarketScan® claims database analysis by Kirsch et al. was conducted among a cohort of CLBP patients with high healthcare utilization (top quartile) who were not surgical candidates (n = 4229) and provided a detailed analysis of healthcare costs and utilization for this patient population over a five year period [18]. The median cost in the first year following diagnosis was roughly $4,405, which is slightly lower than the adjusted cost from our Category 0 cohort described above ($5976) [18]. There were notable differences in our population versus the MarketScan population including more female patients in our cohort (68.4 % vs 56.2 %) and higher rates of depression (38.3 % vs 10.1 %), COPD (24.2 % vs 15.9 %), and obesity (20.5 % vs 8.5 %) compared to the MarketScan population [18]. Additionally, estimates in our study reflect all-cause healthcare charges while CLBP-related costs were reported in the MarketScan population. Another study conducted using a similar CLBP patient population from MarketScan by Spears et al. found all-cause healthcare costs in the first year following diagnosis to be around $6590 [19].

This study correlates with previous literature and corroborates the findings that patients with more severe mechanical CLBP consume the highest HRU and continue to do so over time [18]. Available pharmacological and non-pharmacological treatments often lack the durability to affect this health care utilization trajectory [20]. Surgical intervention when used as a last resort for patients who fail the non-operative treatment options consume even more health care resources and fail to receive durable benefit in addressing their pain [21]. Strategies that durably improve health outcomes inevitably drive down costs long-term, and innovations that can provide this benefit are now needed more than ever [22]. In this respect, we believe the estimated charges could serve as a tool for payers, health systems, and other stakeholders to inform decision-making regarding emerging CLBP therapies.

### 4.1 PROMIS-PF scores

The regression model was centered around patient-reported PROMIS-PF scores as a marker of CLBP severity. In the preliminary stage of data extraction, candidate PROs for our study included the Oswestry Disability Index (ODI) and Pain Visual Analogue Score (PAIN-VAS) as well. However, ODI and PAIN-VAS scores were not consistently stored as structured variables within the database used for this project. Ultimately PROMIS-PF was selected in this study due to 1) notably higher availability of PROMIS-PF scores in our study population, and 2) strong backing in the literature supporting the favorability of PROMIS-PF to other commonly used PROs [12,[23], [24], [25]]. Previous studies have shown that PROMIS-PF outperformed ODI and Short Form-36 (SF-36) in respect to the tests fit, dimensionality, reliability and coverage [23]. Nonetheless, our results can be extrapolated to other patient-reported scoring tools for CLBP using published crosswalks where available. For example, Brodke et al. developed an equation to translate PROMIS-PF scores to and from ODI [26]. The Low-PF (Category 0–1) cohort in this study included PROMIS-PF scores of <38.5, which can be roughly correlated to ODI scores of >37.571 using the equation referenced above. The decision to use the threshold of 38.5 to define our High and Low-PF cohorts was made after preliminary HRU and clinical findings showed a high level of similarity between PROMIS-PF Category 0 and 1 groups, and PROMIS-PF Category 2 and 3 patients.

### 4.2 Pharmacological therapy and procedures

Opioids showed significantly higher utilization among the Low-PF patients compared to High-PF, which has been shown in previous studies [27]. This was consistent across all medication groups except for musculoskeletal agents and anti-inflammatories (non-NSAID). Importantly, the pharmacologic therapy data captured in this study was obtained from EHR medication orders and does not indicate an actual fill as would a prescription claim. As such, medication data here may be under or over-reported as patients may not be filling their medications or may have prescriptions from providers outside of the university-based health system that are not being captured. Nonetheless, claims data includes its own limitations as patients may purchase opioids outside their prescription drug benefit and pay cash, which would bypass the claims system.

Rates of epidural steroid injections and physical therapy were lower than expected in our cohort compared to previous reports [18]. For epidural steroid injections, we speculate this is due to the exclusion of patients with radiculopathy, as they are rarely used in patients without radicular pain within our university-based health system. Physical therapy rates are believed to be under-reported as many patients will receive a referral to an outside clinic to receive therapy, which would not be captured through our university-based database.

Our study followed criteria that excluded surgical candidates within the year prior to and post index date. However, there was a small group of patients who went on to receive costly CLBP-related surgical interventions after the first year of follow-up, despite receiving pharmacologic therapy and other interventional procedures. This highlights the limited number of effective treatment options available and the potential long-term cost implications of these patients with CLBP [28].

### 4.3 Limitations

Our study has several limitations that should be considered when interpreting these results. This was an observational study using real-world data from an EHR database and is subject to missing data and input error on the part of the healthcare provider. While all available PROMIS-PF scores over the two-year follow-up were collected, it is expected that a proportion of the population did not receive an updated score despite a decrease/increase in functionality due to poor follow-up or declining to retake the questionnaire. Further, the regression model used in this study cannot be interpreted as a validated predictive model as it is restricted to a single institution and has not undergone sensitivity analyses to quantify uncertainty. As stated previously, charges are presented here which are proxies of actual cost values. While a CCR can provide an estimate for the cost, it does not reflect the actual costs incurred by this patient population. Additionally, our database is specific to the integrated university-based health system and does not capture care received within hospitals, clinics, or pharmacies outside of the university-based health system. Further, most patients are from one geographic region of the US and may not be generalizable to other populations. The relatively low percentages of non-White patients in this population introduce error into some of the modeled assumptions on healthcare charges for Black and Asian patients given the small sample sizes observed. Similarly, this study aimed to capture a specific subset of mechanical CLBP patients who had who were not candidates for surgical interventions, and therefore our findings may not be applicable to all patients with CLBP. Lastly, this study assessed charges and utilization up through 2 years following diagnosis of CLBP, our results cannot be used to estimate charges beyond this time point.

### 4.4 Implications for future research

Our study quantified the association between PROMIS-PF levels to both healthcare charges and healthcare resource utilization. The implication of these results is that by improving a patient's level of physical function with effective treatment options, one may decrease the consumption of healthcare resources. However, the prospective impacts of either improvement or deterioration in physical function scores at the patient level have not been assessed. Future research on this topic would provide additional evidence on the value of different treatment strategies for patients with mechanical CLBP. Additionally, validation of our findings within an external data set is warranted.

## 5 Conclusion

This study estimated healthcare charges for mechanical CLBP patients based on PROMIS-PF scores. The estimated cumulative 2-year charges were over 2-fold higher among Low Physical Function patients compared to High Physical Function, with significantly higher rates of all-cause inpatient, outpatient, and ED visits in the year following diagnosis for Low Physical Function patients. The disparate healthcare utilization between Low and High Physical Function patients identified in this study combined with clinical outcome data from other trials should inform value assessments of therapeutic options for this difficult to treat patient population.

## Funding disclosure statement

This study received funding support from Mainstay Medical, which is marketing a restorative neurostimulation medical device (ReActiv8) used in to treat mechanical chronic low back pain.

## Ethics statement

This study was approved by the University of Utah IRB_00166316 with an exempt status.

## Funding source

Research funding for this study was provided by Mainstay Medical to the Pharmacotherapy Outcomes Research Center at the 10.13039/100007747University of Utah. The study sponsor participated in protocol and manuscript development.

## Declaration of competing interest

The authors declare the following financial interests/personal relationships which may be considered as potential competing interests:Connor Willis, Andre Hejazi, Xiangyang Ye, Zachary McCormick, & Diana Brixner reports financial support was provided by Mainstay Medical. Connor Willis reports a relationship with 10.13039/100001003Boehringer Ingelheim Pharmaceuticals Inc that includes: funding grants. Connor Willis reports a relationship with 10.13039/100005564Gilead Sciences Inc that includes: funding grants. Connor Willis reports a relationship with 10.13039/100007659Bayer Corporation that includes: funding grants. Connor Willis reports a relationship with 10.13039/100004325AstraZeneca Pharmaceuticals LP that includes: funding grants. Andre Hejazi reports a relationship with 10.13039/100007659Bayer Corporation that includes: funding grants. Andre Hejazi reports a relationship with 10.13039/100014593Neurocrine Biosciences Inc that includes: funding grants. Jim Youssef reports a relationship with Mainstay Medical that includes: employment and equity or stocks. Jim Youssef reports a relationship with Theracell that includes: equity or stocks. Jim Youssef reports a relationship with SeeAll that includes: equity or stocks. Jim Youssef reports a relationship with DermOQ that includes: equity or stocks. Jim Youssef reports a relationship with Globus Medical that includes: consulting, royalty receipts and stock ownership. Chip Moebus reports a relationship with Mainstay Medical Limited that includes: employment and equity or stocks. Benjamin Goss reports a relationship with Mainstay Medical that includes: employment and equity or stocks. Bryan Cornwall reports a relationship with Mainstay Medical that includes: employment, equity or stocks, and travel reimbursement. Darrel Brodke reports a relationship with Orthofix Medical Inc that includes: consulting or advisory. Zachary McCormick reports a relationship with Avanos Medical Inc that includes: funding grants. Zachary McCormick reports a relationship with 10.13039/100008497Boston Scientific Corporation that includes: funding grants. Zachary McCormick reports a relationship with 10.13039/100019095Relievant Medsystems Inc that includes: funding grants. Zachary McCormick reports a relationship with Spine Biopharma that includes: funding grants. Zachary McCormick reports a relationship with 10.13039/100019735SPR Therapeutics Inc that includes: funding grants. Diana Brixner reports a relationship with Sanofi-Aventis US LLC that includes: consulting or advisory. Diana Brixner reports a relationship with 10.13039/501100007132Otsuka Pharmaceutical Co Ltd that includes: consulting or advisory and funding grants. Diana Brixner reports a relationship with Genentech Inc that includes: consulting or advisory and travel reimbursement. Diana Brixner reports a relationship with Gilead Sciences Inc that includes: consulting or advisory and travel reimbursement. Diana Brixner reports a relationship with Lumanity Inc that includes: consulting or advisory. Diana Brixner reports a relationship with 10.13039/100007659Bayer Corporation that includes: funding grants. Diana Brixner reports a relationship with 10.13039/100015769Dexcom Inc that includes: funding grants. Diana Brixner reports a relationship with 10.13039/100004325AstraZeneca Pharmaceuticals LP that includes: funding grants. Diana Brixner reports a relationship with 10.13039/100020337SpringWorks Therapeutics that includes: funding grants. Jim Youseff reports a relationship with Orthofix Medical Inc that includes: equity or stocks. Jim Youseff reports a relationship with CTL-Amedica that includes: equity or stocks. If there are other authors, they declare that they have no known competing financial interests or personal relationships that could have appeared to influence the work reported in this paper.
