# Supplementary material for: The relationship of PROMIS physical function scores and healthcare resource utilization in patients treated for chronic low back pain
Source: Interv Pain Med. 2024 Nov 14;3(4):100522. doi: 10.1016/j.inpm.2024.100522 (PMC11609540; doi:10.1016/j.inpm.2024.100522)
Supplement: Multimedia component 1 [file mmc1.docx]

# Supplementary Materials

**Supplementary Figure 1. Cohort selection flow diagram**


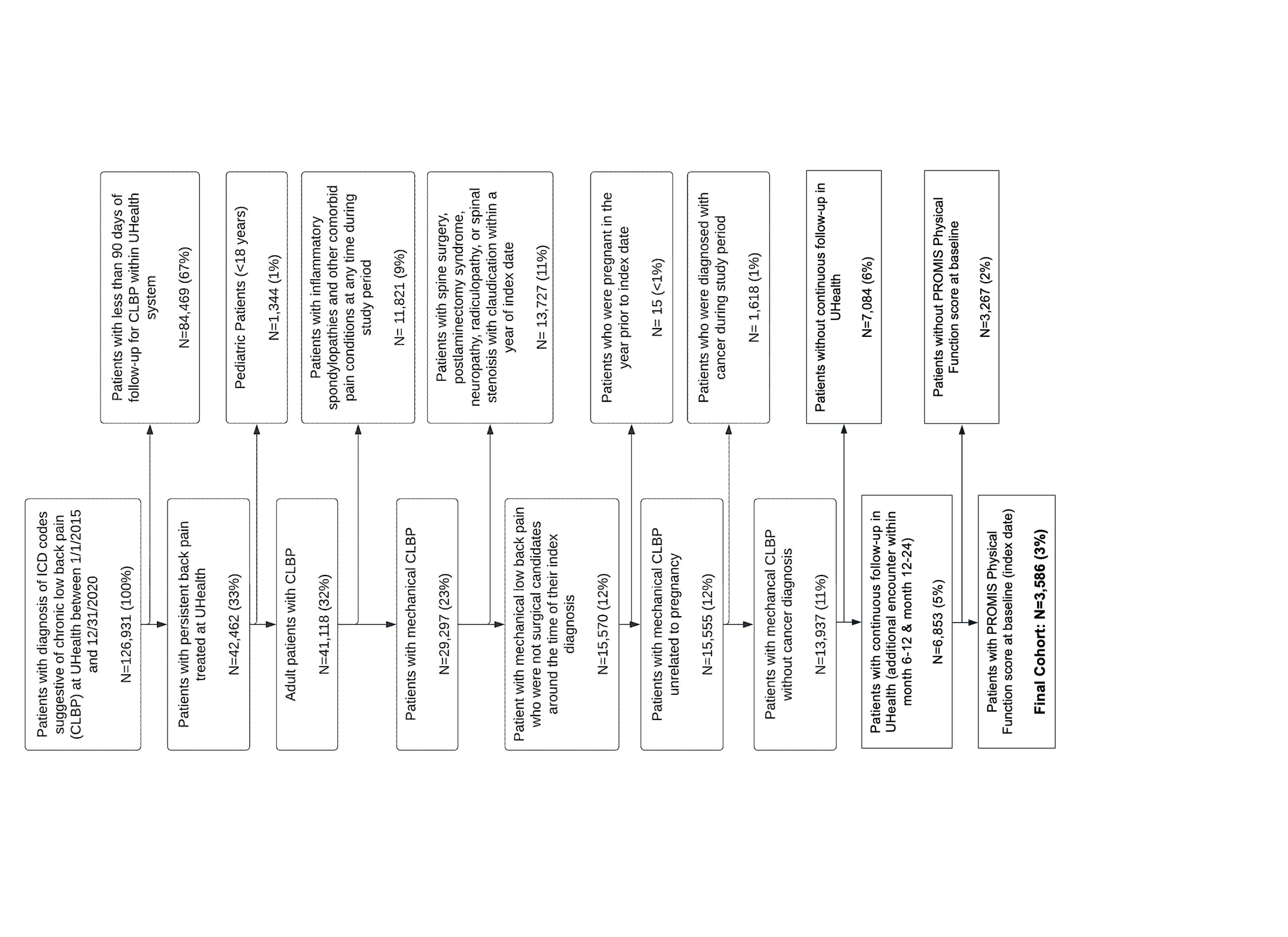


**Supplementary Figure 2. Comparison of predicted Q1 charges for overall cohort between patients with psychological comorbidities**

**
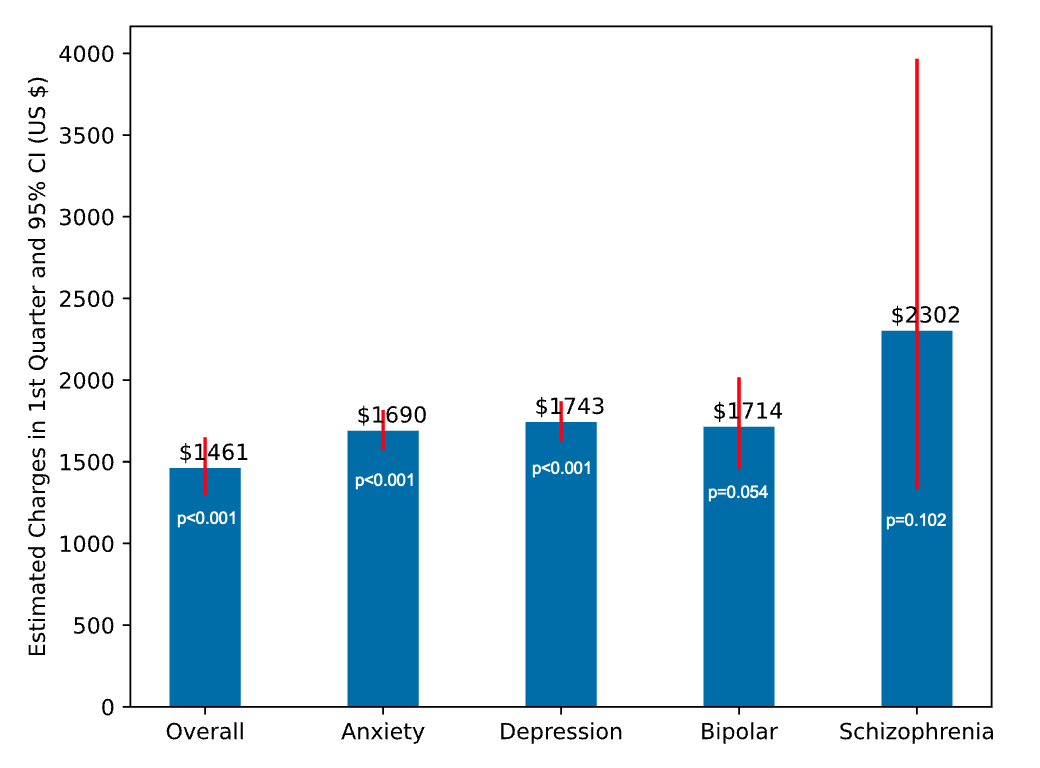
**

Note: these first quarter charges were estimated from predictions of regression models (adjusted for demographic and comorbidities. The red bars represent the 95% confidence intervals (CI).

## Supplementary Table 1. CPT and additional codes

| **CPT codes for non-pharmacologic treatments** | | |
| --- | --- | --- |
| Epidural/facet steroid injection |  | 62310, 62311, 64483, 64484, 64490, 64491, 64492, 64493, 64494, 64495 (CPT) |
| Sympathetic blocks |  | 64505, 64510, 64520, 64530, 64405, 64450, 64418, 64420, 64421, 64425, 64400, 64517, 64455, 64999, 64408 (CPT) |
| Intrathecal pain pumps |  | 62350, 62351, 62355, 62362 (CPT) |
| Ketamine infusions |  | 96365, 96366, 96367, 96368 (CPT) J3490 (HCPCS) |
| Lidocaine infusions |  | J2001 (HCPCS) |
| Trigger point injections |  | 20552, 20553 (CPT) |
| Botulinum toxin injections |  | J0585, J0587, 95874, 64614, 64615 (CPT) |
| Radiofrequency ablations |  | 64633, 64634, 64635, 64636, (CPT) |
| Basivertebral nerve ablation* |  | 64628, 64629, 22899 (Unlisted procedure, spine), 64999 (Unlisted procedure, nervous system) |
| Lower Extremity and Paraspinal EMG |  | 95860 (Needle EMG: 1 extremity with or without paraspinal areas) 95861 (Needle EMG: 2 extremities with or without paraspinal areas) 95863 (Needle EMG: 3 extremities with or without paraspinal areas) 95864 (Needle EMG: 4 extremities with or without paraspinal areas) 95870 (Needle EMG: Limited study of muscles in 1 extremity or non-limb axial muscles (unilateral or bilateral)) other than thoracic paraspinal, cranial nerve supplied muscles or sphincters.) 95873 (Electrical Stimulation for guidance in conjunction with chemodenervation (listed separately in addition to primary procedure)) |
| Acupuncture |  | 97810, 97811, 97813, 97814 (CPT) |
| Physical therapy |  | V57.1 (ICD-9), Z51.89 (ICD-10), 97001, 97002, 97110, 97140, 97124, 97150, 90901, 95831, 95851, 95852, 97010, 97032, 97112, 97116, 97530, 96535, 97161, 97172, 97012, 97014, 97016, 97022, 97024, 97026, 97028, 97032, 97035, 97039, 97110, 97113, 97164, 97533, 97112, 97162, 97163 (CPT) |
| Biofeedback |  | 90875, 90876, 90901, 90911, 95999, 97770, 97112, 97750, 99090, 9081X (CPT) |
| Psychological therapy |  | 90791, 90792, 90832, 90834, 90837, 90801, 90802, 90816, 90818, 90821, 90853, 96150, 96151, 96152, 96153, 97770 (CPT) |
| Chiropractor therapy |  | 98940, 98941, 98942, 98943 (CPT) |
| Massage therapy |  | 97124 (CPT) |
| Occupational therapy |  | 97003, 97004 (CPT) |
| *To supplement unlisted codes patient-level EHR text search will be used using keywords “basivertebral” and “intraosseous” | | |
| **Codes for spine surgeries** | | |
| **Lumbar procedures** | **CPT codes** | **Descriptions** |
| Arthrodesis/fusion | 22612 | Arthrodesis, posterior or posterolateral technique, single level |
| Arthrodesis/fusion + discectomy | 22533 | Arthrodesis, lateral extracavitary technique, including minimal discectomy to prepare interspace (other than for decompression); lumbar |
|  | 22558 | Arthrodesis, anterior interbody technique, including minimal discectomy to prepare interspace (other than for decompression); lumbar |
|  | 0195T (WARNING: code deleted 01/01/19, see 22899) | Arthrodesis, pre-sacral interbody technique, disc space preparation, discectomy, without instrumentation, with image guidance, includes bone graft when performed; L5-S1 interspace |
|  | 0196T (WARNING: code deleted 01/01/19, see 22899) | Arthrodesis, pre-sacral interbody technique, L4-L5 interspace (List separately in addition to code for primary procedure) |
|  | 0309T (WARNING: code deleted 01/01/18, see 22899) | Arthrodesis, pre-sacral interbody technique, including disc space preparation, discectomy, with posterior instrumentation, with image guidance, includes bone graft, when performed, lumbar, L4-L5 interspace (List separately in addition to code for primary procedure) |
| Arthrodesis/fusion + discectomy or laminectomy | 22630 | Arthrodesis, posterior interbody technique, including laminectomy and/or discectomy to prepare interspace (other than for decompression), single interspace; lumbar |
|  | 22633 | Arthrodesis, combined posterior or posterolateral technique with posterior interbody technique including laminectomy and/or discectomy sufficient to prepare interspace (other than for decompression), single interspace and segment; lumbar |
| Corpectomy | 63087 | Vertebral corpectomy (vertebral body resection), partial or complete, combined thoracolumbar approach with decompression of spinal cord, cauda equina or nerve root(s), lower thoracic or lumbar; single segment |
|  | 63088 | Vertebral corpectomy (vertebral body resection), partial or complete, combined thoracolumbar approach with decompression of spinal cord, cauda equina or nerve root(s), lower thoracic or lumbar; each additional segment (List separately in addition to code for primary procedure) |
|  | 63090 | Vertebral corpectomy (vertebral body resection), partial or complete, transperitoneal or retroperitoneal approach with decompression of spinal cord, cauda equina or nerve root(s), lower thoracic, lumbar, or sacral; single segment |
|  | 63091 | Vertebral corpectomy (vertebral body resection), partial or complete, transperitoneal or retroperitoneal approach with decompression of spinal cord, cauda equina or nerve root(s), lower thoracic, lumbar, or sacral; each additional segment (List separately in addition to code for primary procedure) |
|  | 63102 | Vertebral corpectomy (vertebral body resection), partial or complete, lateral extracavitary approach with decompression of spinal cord and/or nerve root(s) (eg, for tumor or retropulsed bone fragments); lumbar, single segment |
|  | 63103 | Vertebral corpectomy (vertebral body resection), partial or complete, lateral extracavitary approach with decompression of spinal cord and/or nerve root(s) (eg, for tumor or retropulsed bone fragments); thoracic or lumbar, each additional segment (List separately in addition to code for primary procedure) |
|  | 63303 | Vertebral corpectomy (vertebral body resection), partial or complete, for excision of intraspinal lesion, single segment; extradural, lumbar or sacral by transperitoneal or retroperitoneal approach |
|  | 63307 | Vertebral corpectomy (vertebral body resection), partial or complete, for excision of intraspinal lesion, single segment; intradural, lumbar or sacral by transperitoneal or retroperitoneal approach |
| Decompression | 63056 | Transpedicular approach with decompression of spinal cord, equina and/or nerve root(s) (eg, herniated intervertebral disc), single segment; lumbar (including transfacet, or lateral extraforaminal approach) (eg, far lateral herniated intervertebral disc) |
|  | 63057 | Transpedicular approach with decompression of spinal cord, equina and/or nerve root(s) (eg, herniated intervertebral disc), single segment; each additional segment, thoracic or lumbar (List separately in addition to code for primary procedure) |
| Decompression + stabilization | 22867 | Insertion of interlaminar/interspinous process stabilization/distraction device, without fusion, including image guidance when performed, with open decompression, lumbar; single level |
|  | 22868 | Insertion of interlaminar/interspinous process stabilization/distraction device, without fusion, including image guidance when performed, with open decompression, lumbar; second level (List separately in addition to code for primary procedure) |
|  | 62380 | Endoscopic decompression of spinal cord, nerve root(s), including laminotomy, partial facetectomy, foraminotomy, discectomy and/or excision of herniated intervertebral disc, 1 interspace, lumbar |
| Disc arthroplasty removal | 22865 | Removal of total disc arthroplasty (artificial disc), anterior approach, single interspace; lumbar |
| Disc arthroplasty revision | 22862 | Revision including replacement of total disc arthroplasty (artificial disc), anterior approach, single interspace; lumbar |
| Laminectomy | 63005 | Laminectomy with exploration and/or decompression of spinal cord and/or cauda equina, without facetectomy, foraminotomy or discectomy (eg, spinal stenosis), 1 or 2 vertebral segments; lumbar, except for spondylolisthesis |
|  | 63012 | Laminectomy with removal of abnormal facets and/or pars inter-articularis with decompression of cauda equina and nerve roots for spondylolisthesis, lumbar (Gill type procedure) |
|  | 63017 | Laminectomy with exploration and/or decompression of spinal cord and/or cauda equina, without facetectomy, foraminotomy or discectomy (eg, spinal stenosis), more than 2 vertebral segments; lumbar |
|  | 63030 | Laminotomy (hemilaminectomy), with decompression of nerve root(s), including partial facetectomy, foraminotomy and/or excision of herniated intervertebral disc; 1 interspace, lumbar |
|  | 63035 | Laminotomy (hemilaminectomy), with decompression of nerve root(s), including partial facetectomy, foraminotomy and/or excision of herniated intervertebral disc; each additional interspace, cervical or lumbar (List separately in addition to code for primary procedure) |
|  | 63047 | Laminectomy, facetectomy and foraminotomy (unilateral or bilateral with decompression of spinal cord, cauda equina and/or nerve root[s], [eg, spinal or lateral recess stenosis]), single vertebral segment; lumbar |
|  | 63048 | Laminectomy, facetectomy and foraminotomy (unilateral or bilateral with decompression of spinal cord, cauda equina and/or nerve root[s], [eg, spinal or lateral recess stenosis]), single vertebral segment; each additional segment, cervical, thoracic, or lumbar (List separately in addition to code for primary procedure) |
|  | 63170 | Laminectomy with myelotomy (eg, Bischof or DREZ type), cervical, thoracic, or thoracolumbar |
|  | 63200 | Laminectomy, with release of tethered spinal cord, lumbar |
|  | 63252 | Laminectomy for excision or occlusion of arteriovenous malformation of spinal cord; thoracolumbar |
|  | 63267 | Laminectomy for excision or evacuation of intraspinal lesion other than neoplasm, extradural; lumbar |
|  | 63272 | Laminectomy for excision of intraspinal lesion other than neoplasm, intradural; lumbar |
| Laminotomy | 63042 | Laminotomy (hemilaminectomy), with decompression of nerve root(s), including partial facetectomy, foraminotomy and/or excision of herniated intervertebral disc, reexploration, single interspace; lumbar |
|  | 63044 | Laminotomy (hemilaminectomy), with decompression of nerve root(s), including partial facetectomy, foraminotomy and/or excision of herniated intervertebral disc, reexploration, single interspace; each additional lumbar interspace (List separately in addition to code for primary procedure) |
| Osteotomy | 22207 | Osteotomy of spine, posterior or posterolateral approach, 3 columns, 1 vertebral segment (eg, pedicle/vertebral body subtraction); lumbar |
|  | 22214 | Osteotomy of spine, posterior or posterolateral approach, 1 vertebral segment; lumbar |
|  | 22224 | Osteotomy of spine, including discectomy, anterior approach, single vertebral segment; lumbar |
| Partial excision of vertebral body | 22114 | Partial excision of vertebral body, for intrinsic bony lesion, without decompression of spinal cord or nerve root(s), single vertebral segment; lumbar |
| Particial excision of vertebral component | 22102 | Partial excision of posterior vertebral component (eg, spinous process, lamina or facet) for intrinsic bony lesion, single vertebral segment; lumbar |
| Stabilization | 22869 | Insertion of interlaminar/interspinous process stabilization/distraction device, without open decompression or fusion, including image guidance when performed, lumbar; single level |
|  | 22870 | Insertion of interlaminar/interspinous process stabilization/distraction device, without open decompression or fusion, including image guidance when performed, lumbar; second level (List separately in addition to code for primary procedure) |
| Total disc arthroplasty and discectomy | 22857 | Total disc arthroplasty (artificial disc), anterior approach, including discectomy to prepare interspace (other than for decompression), single interspace, lumbar |
| **Sacral procedures** | **CPT codes** | **Descriptions** |
| Arthrodesis/fusion | 22586 | Arthrodesis, pre-sacral interbody technique, including disc space preparation, discectomy, with posterior instrumentation, with image guidance, includes bone graft when performed, L5-S1 interspace |
| Corpectomy | 63090 | Vertebral corpectomy (vertebral body resection), partial or complete, transperitoneal or retroperitoneal approach with decompression of spinal cord, cauda equina or nerve root(s), lower thoracic, lumbar, or sacral; single segment |
|  | 63091 | Vertebral corpectomy (vertebral body resection), partial or complete, transperitoneal or retroperitoneal approach with decompression of spinal cord, cauda equina or nerve root(s), lower thoracic, lumbar, or sacral; each additional segment (List separately in addition to code for primary procedure) |
| Laminectomy | 63011 | Laminectomy with exploration and/or decompression of spinal cord and/or cauda equina, without facetectomy, foraminotomy or discectomy (eg, spinal stenosis), 1 or 2 vertebral segments; sacral |
|  | 63268 | Laminectomy for excision or evacuation of intraspinal lesion other than neoplasm, extradural; sacral |
|  | 63273 | Laminectomy for excision of intraspinal lesion other than neoplasm, intradural; sacral |
| **Unspecified Procedures** | **CPT codes** | **Descriptions** |
| Arthrodesis/fusion | 22614 | Arthrodesis, posterior or posterolateral technique, single level; each additional vertebral segment (List separately in addition to code for primary procedure) |
|  | 22800 | Arthrodesis, posterior, for spinal deformity, with or without cast; up to 6 vertebral segments |
|  | 22802 | Arthrodesis, posterior, for spinal deformity, with or without cast; 7 to 12 vertebral segments |
|  | 22804 | Arthrodesis, posterior, for spinal deformity, with or without cast; 13 or more vertebral segments |
|  | 22808 | Arthrodesis, anterior, for spinal deformity, with or without cast; 2 to 3 vertebral segments |
|  | 22810 | Arthrodesis, anterior, for spinal deformity, with or without cast; 4 to 7 vertebral segments |
|  | 22812 | Arthrodesis, anterior, for spinal deformity, with or without cast; 8 or more vertebral segments |
| Arthrodesis/fusion and discectomy | 22534 | Arthrodesis, lateral extracavitary technique, including minimal discectomy to prepare interspace (other than for decompression); thoracic or lumbar, each additional vertebral segment (List separately in addition to code for primary procedure) |
|  | 22585 | Arthrodesis, anterior interbody technique, including minimal discectomy to prepare interspace (other than for decompression); each additional interspace (List separately in addition to code for primary procedure) |
|  | 22632 | Arthrodesis, posterior interbody technique, including laminectomy and/or discectomy to prepare interspace (other than for decompression), single interspace; each additional interspace (List separately in addition to code for primary procedure) |
|  | 22634 | Arthrodesis, combined posterior or posterolateral technique with posterior interbody technique including laminectomy and/or discectomy sufficient to prepare interspace (other than for decompression), single interspace and segment; each additional interspace and segment (List separately in addition to code for primary procedure) |
|  | 22558 | Arthrodesis, anterior interbody technique, including minimal discectomy to prepare interspace (other than for decompression) |
|  | 22533 | Arthrodesis, lateral extracavitary technique, including minimal discectomy to prepare interspace (other than for decompression) |
| Corpectomy | 63308 | Vertebral corpectomy (vertebral body resection), partial or complete, for excision of intraspinal lesion, single segment; each additional segment (List separately in addition to codes for single segment) |
| Fixation device re-insertion | 22849 | Reinsertion of spinal fixation device |
| Fusion exploration | 22830 | Exploration of spinal fusion |
| Intrumentation | 22850 | Removal of posterior nonsegmental instrumentation (eg, Harrington rod) |
|  | 22842 | Posterior segmental instrumentation (eg, pedicle fixation, dual rods with multiple hooks and sublaminar wires); 3 to 6 vertebral segments (List separately in addition to code for primary procedure) |
|  | 22843 | Posterior segmental instrumentation (eg, pedicle fixation, dual rods with multiple hooks and sublaminar wires); 7 to 12 vertebral segments (List separately in addition to code for primary procedure) |
|  | 22844 | Posterior segmental instrumentation (eg, pedicle fixation, dual rods with multiple hooks and sublaminar wires); 13 or more vertebral segments (List separately in addition to code for primary procedure) |
|  | 22853 | Insertion of interbody biomechanical device(s) (eg, synthetic cage, mesh) with integral anterior instrumentation for device anchoring (eg, screws, flanges), when performed, to intervertebral disc space in conjunction with interbody arthrodesis, each interspace (List separately in addition to code for primary procedure) |
|  | 22854 | Insertion of intervertebral biomechanical device(s) (eg, synthetic cage, mesh) with integral anterior instrumentation for device anchoring (eg, screws, flanges), when performed, to vertebral corpectomy(ies) (vertebral body resection, partial or complete) defect, in conjunction with interbody arthrodesis, each contiguous defect (List separately in addition to code for primary procedure) |
|  | 22859 | Insertion of intervertebral biomechanical device(s) (eg, synthetic cage, mesh, methylmethacrylate) to intervertebral disc space or vertebral body defect without interbody arthrodesis, each contiguous defect (List separately in addition to code for primary procedure) |
|  | 22634 | Arthrodesis, combined posterior or posterolateral technique with posterior interbody technique including laminectomy and/or discectomy sufficient to prepare interspace (other than for decompression), single interspace and segment; lumbar |
| Instrumentation removal | 22840 | Posterior non-segmental instrumentation (eg, Harrington rod technique, pedicle fixation across 1 interspace, atlantoaxial transarticular screw fixation, sublaminar wiring at C1, facet screw fixation) (List separately in addition to code for primary procedure) |
|  | 22852 | Removal of posterior segmental instrumentation |
|  | 22855 | Removal of anterior instrumentation |
| Internal fixation | 22841 | Internal spinal fixation by wiring of spinous processes (List separately in addition to code for primary procedure) |
| Pelvic fixation | 22848 | Pelvic fixation (attachment of caudal end of instrumentation to pelvic bony structures) other than sacrum (List separately in addition to code for primary procedure) |
| Kyphectomy | 22818 | Kyphectomy, circumferential exposure of spine and resection of vertebral segment(s) (including body and posterior elements); single or 2 segments |
|  | 22819 | Kyphectomy, circumferential exposure of spine and resection of vertebral segment(s) (including body and posterior elements); 3 or more segments |
| Laminectomy with rhizotomy | 63185 | Laminectomy with rhizotomy; 1 or 2 segments |
|  | 63190 | Laminectomy with rhizotomy; more than 2 segments |
| Laminectomy with section of spinal accessory nerve | 63191 | Laminectomy with section of spinal accessory nerve |
| Laminotomy | 63042 | Laminotomy (hemilaminectomy), with decompression of nerve root(s), including partial facetectomy, foraminotomy and/or excision of herniated intervertebral disc, reexploration, single interspace |
| Osteoplastic reconstruction following primary procedure | 63295 | Osteoplastic reconstruction of dorsal spinal elements, following primary intraspinal procedure (List separately in addition to code for primary procedure) |
| Ostetomy | 22208 | Osteotomy of spine, posterior or posterolateral approach, 3 columns, 1 vertebral segment (eg, pedicle/vertebral body subtraction); each additional vertebral segment (List separately in addition to code for primary procedure) |
|  | 22216 | Osteotomy of spine, posterior or posterolateral approach, 1 vertebral segment; each additional vertebral segment (List separately in addition to primary procedure) |
|  | 22226 | Osteotomy of spine, including discectomy, anterior approach, single vertebral segment; each additional vertebral segment (List separately in addition to code for primary procedure) |
| Partial excision of vertebral body | 22116 | Partial excision of vertebral body, for intrinsic bony lesion, without decompression of spinal cord or nerve root(s), single vertebral segment; each additional vertebral segment (List separately in addition to code for primary procedure) |
| Partial excision of vertebral component | 22103 | Partial excision of posterior vertebral component (eg, spinous process, lamina or facet) for intrinsic bony lesion, single vertebral segment; each additional segment (List separately in addition to code for primary procedure) |
| **Other codes** |  |  |
| Codes for SCS | Lead implantation | CPT: 63650, 63655 ICD-9-CM: 03.93 ICD-10: A42.2 HCPCS: L8680, C1778, C1897, C1883 |
|  | Pulse generator implantation | CPT: 63685 ICD-9-CM: 86.94-86.98 ICD-10: 0JH60BZ, 0JH63BZ, 0JH70BZ, 0JH73BZ, 0JH80BZ, 0JH83BZ, 0JH60DZ, 0JH63DZ, 0JH70DZ, 0JH73DZ, 0JH80DZ, 0JH83DZ, 0JH60MZ, 0JH63MZ, 0JH70MZ, 0JH73MZ, 0JH80MZ, 0JH83MZ, 0JH60CZ, 0JH63CZ, 0JH70CZ, 0JH73CZ, 0JH80CZ, 0JH83CZ, 0JH60EZ, 0JH63EZ, 0JH70EZ, 0JH73EZ, 0JH80EZ, 0JH83EZ HCPCS: L8685-L8688, C1767, C1820, C1822, L8679, L8682 |
| Codes for post-laminectomy syndrome | ICD-9 ICD-10 | 722.80-722.83, V45.89 M96.1, Z98.89 |
| Codes for failed back surgery syndrome | ICD-10 | Z98.89, M96.82, T84.226A |
|  |  |  |
| **Codes for comorbidities** | | |
| Paralysis |  | G81 , G81.0 , G81.00 , G81.01 , G81.02 , G81.03 , G81.04 , G81.1 , G81.10 , G81.11 , G81.12 , G81.13 , G81.14 , G81.9 , G81.90 , G81.91 , G81.92 , G81.93 , G81.94 , G82 , G82.2 , G82.20 , G82.21 , G82.22 , G82.5 , G82.50 , G82.51 , G82.52 , G82.53 , G82.54 , G83 , G83.0 , G83.1 , G83.10 , G83.11 , G83.12 , G83.13 , G83.14 , G83.2 , G83.20 , G83.21 , G83.22 , G83.33 , G83.34 , G83.4 , G83.5 , G83.8 , G83.81 , G83.82 , G83.83 , G83.84 , G83.89 , G83.9 |
| Coagulopathy |  | D68, D68.0,  D68.00,  D68.01,  D68.02,  D68.020,  D68.021,  D68.022,  D68.023,  D68.029,  D68.03,  D68.04,  D68.09,  D68.1,  D68.2,  D68.3,  D68.31,  D68.311,  D68.312,D68.318,D68.32, D68.4, D68.5, D68.51,  D68.52, D68.59, D68.6, D68.61,  D68.62, D68.69, D68.8, D68.9 |
| Rheumatoid arthritis |  | M06.90, M06.91, M06.92, M06.93, M06.94, M06.95, M06.96, M06.97, M06.98, M06.99, M05 |
| Congestive heart failure |  | I50.2, I50.3, I50.4, I50.41, I50.40, I50.21, I50.22,I50.23, I50.31, I50.32, I50.33, I50.20, I50.30, I50.42 |
| Chronic pulmonary disease |  | J44.9, J44.1, J44, J44.0 |
| HIV/AIDS |  | Z21, B20 |
| Depression |  | F32.0, F32.1, F32.2, F32.3, F32.4, F32.5, F32.8, F32.81, F32.89, F32.9, F32.A, F33, F33.0, F33.1, F33.2, F33.3, F33.4, F33.40, F33.41, F33.42, F33.8 |
| Diabetes without chronic complications |  | E10.9, E11.9, E13.9 |
| Diabetes with chronic complications |  | E10.0, E10.1, E10.2, E10.3, E10.4, E10.5, E10.6, E10.7, E10.8, E11.0, E11.1, E11.2, E11.3, E11.4, E11.5, E11.6, E11.7, E11.8, E13.0, E13.1, E13.2, E13.3, E13.4, E13.5, E13.6, E13.7, E13.8 |
| Substance abuse disorder |  | F19, F19.1, F19.10, F19.11, F19.12, F19.120, F19.121, F19.122, F19.129, F19.13, F19.130 |
| Liver disease |  | K75, , K75.0, , K75.1, , K75.2, , K75.3, , K75.4, , K75.8, , K75.81, , K75.89, , K75.9, K71.9, K71.11, K71.2, , K71.3, , K71.4, , K71.5, , K71.50, , K71.51, , K71.6, , K71.7, , K71.8, , K71.9, , K72, , K72.00, , K72.01, K72.1, , K72.10, , K72.11, , K72.9, , K72.90, , K72.91, K76.2, , K76.3, , K76.4, , K76.5, , K76.6, , K76.7, , K76.8, , K76.81, , K76.82, , K76.89, , K76.9, , K77 |
| Hypothyroidism |  | E03.8, E03.9 |
| Hypertension |  | I10 |
| Obesity |  | E66, E66.0, E66.01, E66.09, E66.1, E66.2, E66.3, E66.8, E66.9 |
| Peripheral vascular disease |  | I73 , I73.0 , I73.00 , I73.01 , I73.1 , I73.8 , I73.81 , I73.89 , I73.9 , |
| Renal failure |  | N17 , N17.0 , N17.1 , N17.2 , N17.8 , N17.9 ,N18 , N18.1 , N18.2 , N18.3 , N18.30 , N18.31 , N18.32 , N18.4 , N18.5 , N18.6 , N18.9 , N19 |
| Peptic ulcer disease |  | K27 , K27.0 , K27.1 , K27.2 , K27.3 , K27.4 , K27.5 , K27.6 , K27.7 , K27.9 |
|  | | |

**Supplementary Table 2. Medications included in classes**

| Opioids: | Buprenorphine, Codeine, Fentanyl, Hydrocodone, Hydromorphone, Meperidine, Methadone, Morphine Sulfate, Nalbuphine, Oxycodone, Oxymorphone, Remifentanil, Sufentanil, Tapentadol, Tramadol |
| --- | --- |
| NSAIDs: | Aspirin, Celecoxib, Diclofenac, Etodolac, Ibuprofen, Indomethacin, Ketorlac, Meloxicam, Nabumetone, Naptroxen, Oxaprozin, Suldinacx |
| Acetaminophen: | Single agent and non-opioid combination formulations |
| Musculoskeletal agents: | Carisoprodol, Chlorzoxazone, Cyclobenzaprine, Metaxalone, Methocarbamol, Orphenadrine, Tizanidine |
| Anticonvulsants: | Carbamazepine, Clobazam, Clonazepam, Diazepam, Divalproex, Ethosuximide, Fosphenytoin, Gabapentin, Lacosamide, Lamotrigine, Levetiracetam, Oxcarbazepine, Phenytoin, Pregabalin, Primidone, Topiramate, Valproic Acid, Zonisamide |
| Antidepressants: | Amitriptyline, Bupropion, Citalopram, Clomipramine, Desipramine, Desvenlafaxine, Doxepin, Duloxetine, Escitalopram, Fluoxetine, Fluvoxamine, Imipramine, Imipramine, Isocarboxazid, Mirtazapine, Nefazodone, Nortriptyline, Paroxetine, Paroxetine, Phenelzine, Protriptyline, Selegiline, Sertraline, Tranylcypromine, Trazodone, Venlafaxine, Vilazodone |

**Supplementary Table 3. Surgical procedures received following 1-year post index date (n=14)**

| **CPT code** | **Description** |
| --- | --- |
| 22633 | Arthrodesis, combined posterior or posterolateral technique with posterior interbody technique including laminectomy and/or discectomy sufficient to prepare interspace (other than for decompression), single interspace and segment; lumbar |
| 22840 | Posterior non-segmental instrumentation (eg, Harrington rod technique, pedicle fixation across 1 interspace, atlantoaxial transarticular screw fixation, sublaminar wiring at C1, facet screw fixation) (List separately in addition to code for primary procedure) |
| 22853 | Insertion of interbody biomechanical device(s) (eg, synthetic cage, mesh) with integral anterior instrumentation for device anchoring (eg, screws, flanges), when performed, to intervertebral disc space in conjunction with interbody arthrodesis, each interspace (List separately in addition to code for primary procedure) |
| 22612 | Arthrodesis, posterior or posterolateral technique, single level |
| 63047 | Laminectomy, facetectomy and foraminotomy (unilateral or bilateral with decompression of spinal cord, cauda equina and/or nerve root[s], [eg, spinal or lateral recess stenosis]), single vertebral segment; lumbar |
| 63048 | Laminectomy, facetectomy and foraminotomy (unilateral or bilateral with decompression of spinal cord, cauda equina and/or nerve root[s], [eg, spinal or lateral recess stenosis]), single vertebral segment; each additional segment, cervical, thoracic, or lumbar (List separately in addition to code for primary procedure) |
| 22614 | Arthrodesis, posterior or posterolateral technique, single level; each additional vertebral segment (List separately in addition to code for primary procedure) |
| 22842 | Posterior segmental instrumentation (eg, pedicle fixation, dual rods with multiple hooks and sublaminar wires); 3 to 6 vertebral segments (List separately in addition to code for primary procedure) |
| 22633 | Arthrodesis, combined posterior or posterolateral technique with posterior interbody technique including laminectomy and/or discectomy sufficient to prepare interspace (other than for decompression), single interspace and segment; lumbar |
| 63030 | Laminotomy (hemilaminectomy), with decompression of nerve root(s), including partial facetectomy, foraminotomy and/or excision of herniated intervertebral disc; 1 interspace, lumbar |
| 22558 | Arthrodesis, anterior interbody technique, including minimal discectomy to prepare interspace (other than for decompression); lumbar |
| 63035 | Laminotomy (hemilaminectomy), with decompression of nerve root(s), including partial facetectomy, foraminotomy and/or excision of herniated intervertebral disc; each additional interspace, cervical or lumbar (List separately in addition to code for primary procedure) |
| 63267 | Laminectomy for excision or evacuation of intraspinal lesion other than neoplasm, extradural; lumbar |
| 22630 | Arthrodesis, posterior interbody technique, including laminectomy and/or discectomy to prepare interspace (other than for decompression), single interspace; lumbar |
| 22632 | Arthrodesis, posterior interbody technique, including laminectomy and/or discectomy to prepare interspace (other than for decompression), single interspace; each additional interspace (List separately in addition to code for primary procedure) |
| 63012 | Laminectomy with removal of abnormal facets and/or pars inter-articularis with decompression of cauda equina and nerve roots for spondylolisthesis, lumbar (Gill type procedure) |
| 22843 | Posterior segmental instrumentation (eg, pedicle fixation, dual rods with multiple hooks and sublaminar wires); 7 to 12 vertebral segments (List separately in addition to code for primary procedure) |
